# Supplementary figures and images for: A complementary approach for detecting biological signals through a semi-automated feature selection tool
Source: Front Chem. 2024 Oct 25;12:1477492. doi: 10.3389/fchem.2024.1477492 (PMC11543558; doi:10.3389/fchem.2024.1477492)

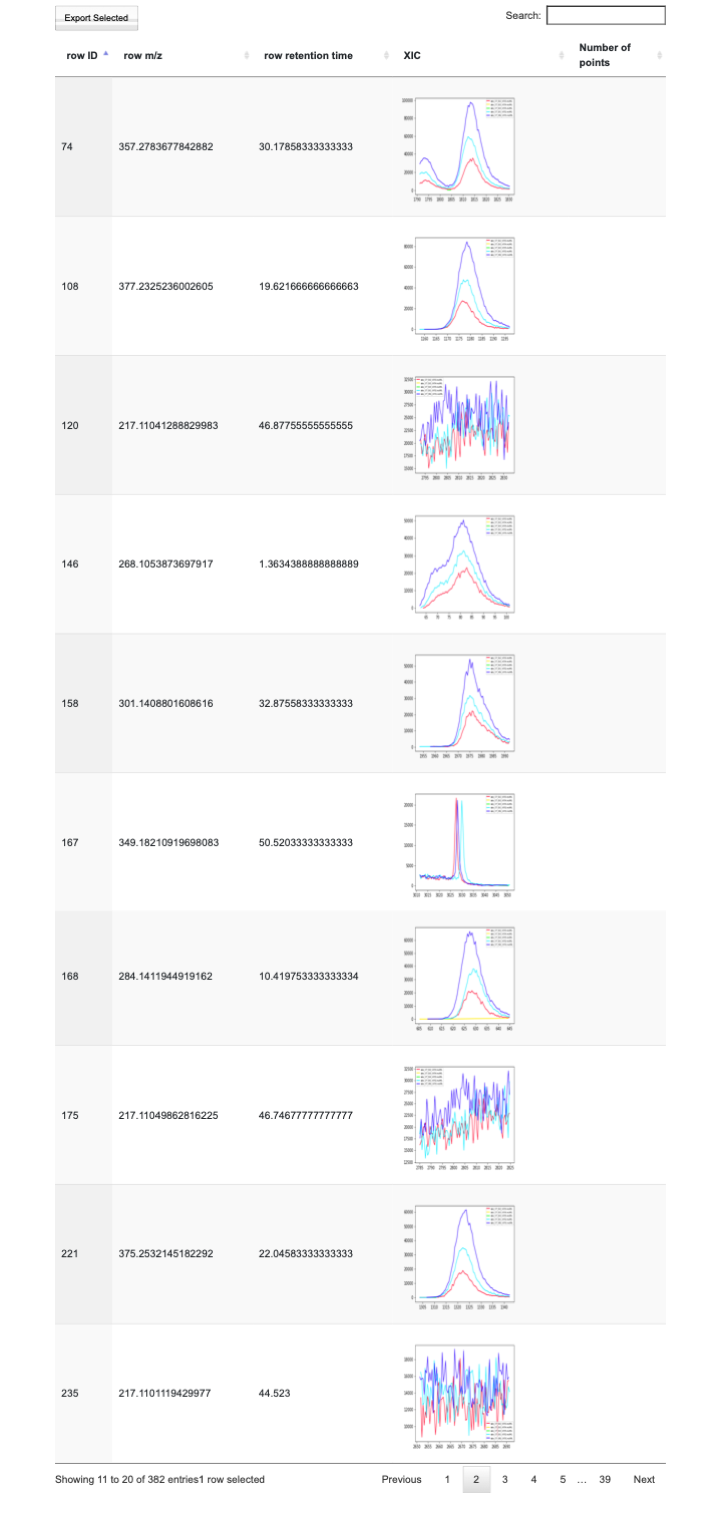

Supplement: Supplementary file 1 [file Image4.PNG]

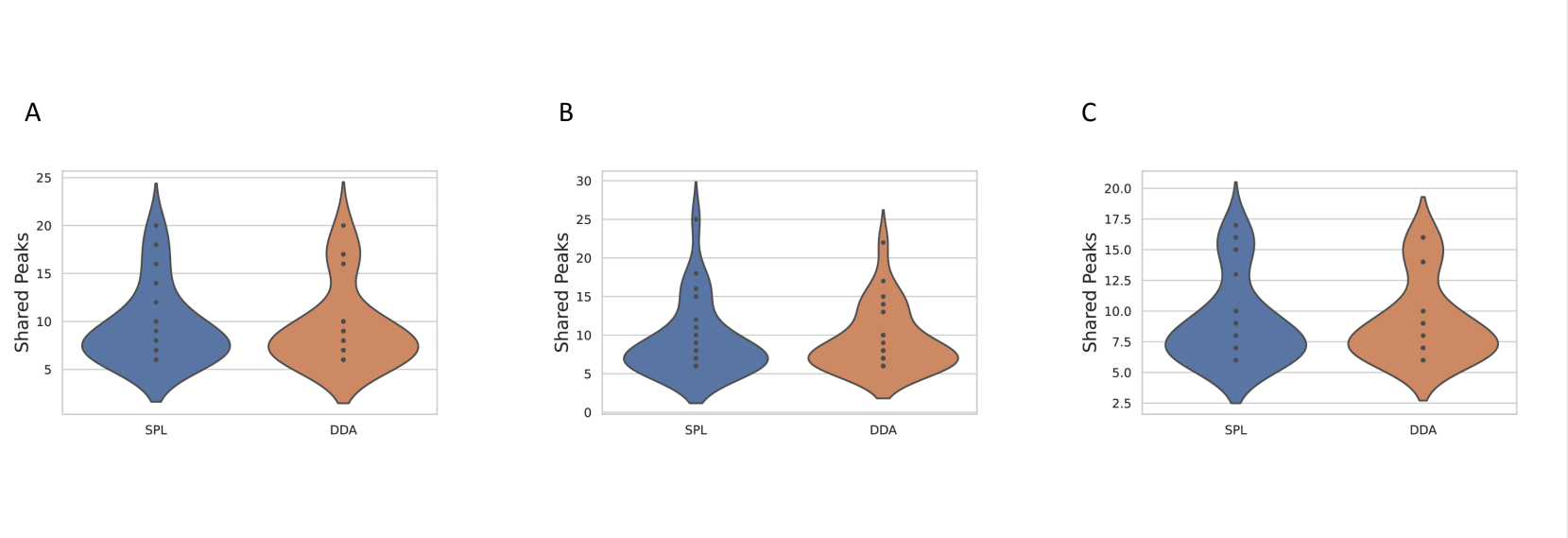

Supplement: Supplementary file 2 [file Image2.PNG]

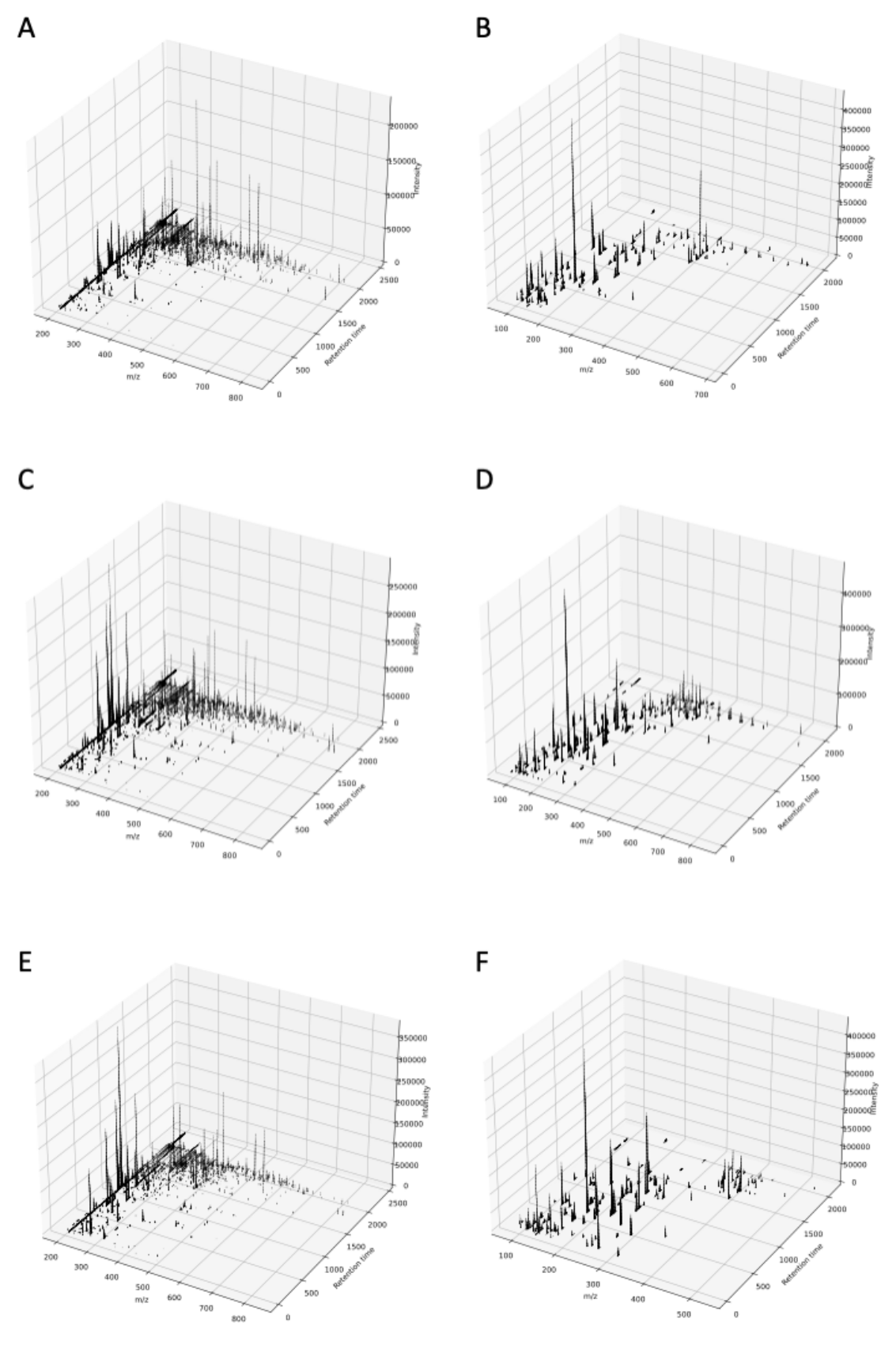

Supplement: Supplementary file 4 [file Image1.PNG]

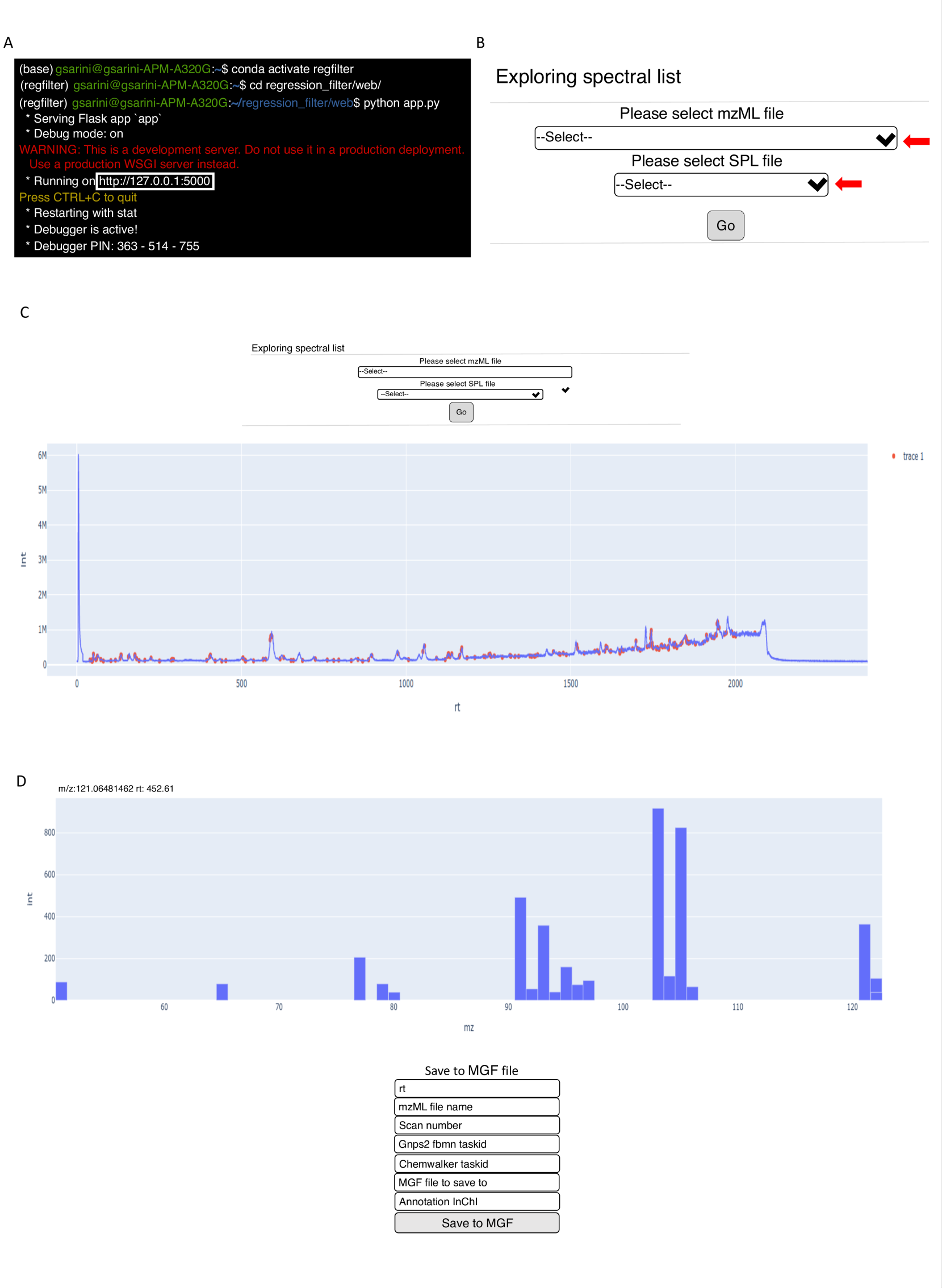

Supplement: Supplementary file 5 [file Image3.PNG]
