# Supplementary material for: A complementary approach for detecting biological signals through a semi-automated feature selection tool
Source: Front Chem. 2024 Oct 25;12:1477492. doi: 10.3389/fchem.2024.1477492 (PMC11543558; doi:10.3389/fchem.2024.1477492)
Supplement: Supplementary file 3 [file DataSheet2.docx]

***Supplementary Material***

Figures

A complementary approach for detecting biological signals through a semi-automated feature selection tool: introducing Regression Filter

Gabriel Santos Arini, Luiz Gabriel Mencucini, Rafael de Felício, Luís Guilherme Pereira Feitosa, Paula Rezende Teixeira, Henrique Tsuji, Alan Pilon, Danielle Rocha Pinho, Letícia Veras Costa Lotufo, Norberto Peporine Lopes, Daniella Barretto Barbosa Trivella, Ricardo Roberto da Silva

Supplementary Figure 1

Supplementary Figure 1. 3D chromatograms for the fragmented precursor ion for the actinomycetes BRA006, BRA010 and BRA177. A, C and E refer to the fragmented precursor ion acquired in DDA for BRA006, BRA010 and BRA177, respectively. B, D and F refer to the fragmented precursor ion acquired in SPL for BRA006, BRA010 and BRA177, respectively.

Supplementary Figure 2

Supplementary Figure 2. Violin plots comparing the average peaks shared by the same precursor ions in SPL and DDA when paired with reference spectra in the GNPS2 library (Wang et al., 2016), namely (A) BRA006, (B) BRA010 and (C) BRA177.

Supplementary Figure 3

Supplementary Figure 3. Step-by-step instructions for creating in-house spectral libraries using RegFilter. After installing RegFilter follow the additional graphical interface instructions (https://github.com/computational-chemical-biology/regression_filter/tree/main/web). By clicking on the link shown in the white box (A), the user is taken to their default web browser. There (B), the user selects the spectrometric data of interest and the desired fragmentation list. Once both have been selected, the spectrum is displayed, with the red dots in the fragmentation list (C) indicating the features included in the list. By clicking on one of the points of interest, the user is presented with the corresponding fragmentation spectrum and can make insertions of interest in the .mgf file to be exported (D).

Supplementary Figure 4

Supplementary Figure 4. Result of the interactive *output* of the Regression Filter for viewing the extracted ion chromatograms (XICs) of the features selected by the tool. It is possible to distinguish a true signal from noise. The true signal has a sinusoidal shape, while the noisy signal has a random aspect.
