# Supplementary material for: A complementary approach for detecting biological signals through a semi-automated feature selection tool
Source: Front Chem. 2024 Oct 25;12:1477492. doi: 10.3389/fchem.2024.1477492 (PMC11543558; doi:10.3389/fchem.2024.1477492)
Supplement: Supplementary file 6 [file DataSheet1.docx]

***Supplementary Material***

Tables

A complementary approach for detecting biological signals through a semi-automated feature selection tool: introducing Regression Filter

Gabriel Santos Arini, Luiz Gabriel Mencucini, Rafael de Felício, Luís Guilherme Pereira Feitosa, Paula Rezende Teixeira, Henrique Tsuji, Alan Pilon, Danielle Rocha Pinho, Letícia Veras Costa Lotufo, Norberto Peporine Lopes, Daniella Barretto Barbosa Trivella, Ricardo Roberto da Silva

Supplementary Table 1. Molecular annotations obtained from the GNPS2, SIRIUS and ChemWalker tools for the features included only in the list for Actinomycetes BRA006, BRA010, and BRA177.

| Sample | Precursor Mass (m/z) | Retention time (min) | Annotated Method | InChIKey | IUPAC name |
| --- | --- | --- | --- | --- | --- |
| BRA006 | 136.076 | 0.05 | ChemWalker | ZXTLGJAARBNQGK-UHFFFAOYSA-N | N-(2-methylphenyl)formamide |
| BRA006 | 108.045 | 0.81 | ChemWalker | CSDSSGBPEUDDEE-UHFFFAOYSA-N | pyridine-2-carbaldehyde |
| BRA006 | 96.045 | 0.82 | ChemWalker | ZSKGQVFRTSEPJT-UHFFFAOYSA-N | 1H-pyrrole-2-carbaldehyde |
| BRA006 | 126.055 | 0.83 | ChemWalker | VJBRLHBYLMMWER-UHFFFAOYSA-N | N-[(5-methylfuran-2-yl)methylidene]hydroxylamine |
| BRA006 | 110.060 | 0.83 | ChemWalker | OUKQTRFCDKSEPL-UHFFFAOYSA-N | 1-methylpyrrole-2-carbaldehyde |
| BRA006 | 167.093 | 0.84 | SIRIUS | PVWNFAGYFUUDRC-UHFFFAOYSA-N | N-[(4-amino-2-methylpyrimidin-5-yl)methyl]formamide |
| BRA006 | 123.047 | 1.09 | ChemWalker | YODZTKMDCQEPHD-UHFFFAOYSA-N | 2-(2-hydroxyethylsulfanyl)ethanol |
| BRA006 | 137.071 | 1.37 | ChemWalker | RHPIFAMEPPCHGX-UHFFFAOYSA-N | 6-(hydrazinylmethylidene)cyclohexa-2,4-dien-1-one |
| BRA006 | 153.041 | 1.57 | GNPS2 | LRFVTYWOQMYALW-UHFFFAOYSA-N | 3,7-dihydropurine-2,6-dione |
| BRA006 | 174.055 | 2.06 | SIRIUS | GZVIFFZHYPUWML-UHFFFAOYSA-N | N-[2-(2-hydroxyethoxy)ethyl]carbamoyl fluoride |
| BRA006 | 155.082 | 2.22 | ChemWalker | OWOHLURDBZHNGG-UHFFFAOYSA-N | 2,3,6,7,8,8a-hexahydropyrrolo[1,2-a]pyrazine-1,4-dione |
| BRA006 | 111.056 | 2.42 | ChemWalker | RLOQBKJCOAXOLR-UHFFFAOYSA-N | 1H-pyrrole-2-carboxamide |
| BRA006 | 160.076 | 2.56 | ChemWalker | WHOOUMGHGSPMGR-UHFFFAOYSA-N | 2-(1H-indol-3-yl)acetaldehyde |
| BRA006 | 112.040 | 2.59 | ChemWalker | TVFIYRKPCACCNL-UHFFFAOYSA-N | furan-2-carboxamide |
| BRA006 | 164.093 | 2.63 | ChemWalker | BVIAOQMSVZHOJM-UHFFFAOYSA-N | N,N-dimethyl-7H-purin-6-amine |
| BRA006 | 111.045 | 2.95 | ChemWalker | UBTPQJCPHOHKMF-UHFFFAOYSA-N | 5-ethylidenefuran-2-one |
| BRA006 | 138.055 | 3.02 | GNPS2 | LOIYMIARKYCTBW-UHFFFAOYSA-N | 3-(1H-imidazol-5-yl)prop-2-enoic acid |
| BRA006 | 157.097 | 3.08 | SIRIUS | ZHDUPSJIKMSXIY-UHFFFAOYSA-N | N-methyl-2-(2-oxopyrrolidin-1-yl)acetamide |
| BRA006 | 152.071 | 3.46 | GNPS2 | CCVYRRGZDBSHFU-UHFFFAOYSA-N | 2-(2-hydroxyphenyl)acetic acid |
| BRA006 | 100.076 | 3.98 | SIRIUS | ZAEGKNQQHQDJKF-UHFFFAOYSA-O | pentanamide |
| BRA006 | 112.022 | 4.83 | SIRIUS | NIPDFDJOGCAWGH-UHFFFAOYSA-N | methoxy(methyl)borinic acid |
| BRA006 | 130.032 | 4.86 | SIRIUS | YNSUBIQKQNBKJT-UHFFFAOYSA-N | 2-(1,3-thiazol-2-yl)ethanol |
| BRA006 | 168.066 | 4.99 | GNPS2 | WKOLLVMJNQIZCI-UHFFFAOYSA-N | 4-hydroxy-3-methoxybenzoic acid |
| BRA006 | 152.071 | 6.40 | GNPS2 | SULYEHHGGXARJS-UHFFFAOYSA-N | 1-(2,4-dihydroxyphenyl)ethanone |
| BRA006 | 122.060 | 6.65 | GNPS2 | KXDAEFPNCMNJSK-UHFFFAOYSA-N | benzamide |
| BRA006 | 171.113 | 7.47 | ChemWalker | GINVVVBYRCSQIJ-UHFFFAOYSA-N | 2-(2-oxoazepan-1-yl)acetamide |
| BRA006 | 171.113 | 7.51 | ChemWalker | GINVVVBYRCSQIJ-UHFFFAOYSA-N | 2-(2-oxoazepan-1-yl)acetamide |
| BRA006 | 158.059 | 8.36 | ChemWalker | VUAOIXANWIFYCU-UHFFFAOYSA-N | quinoline-6-carbaldehyde |
| BRA006 | 121.065 | 9.78 | ChemWalker | BTFQKIATRPGRBS-UHFFFAOYSA-N | 2-methylbenzaldehyde |
| BRA006 | 103.054 | 9.80 | ChemWalker | UEXCJVNBTNXOEH-UHFFFAOYSA-N | ethynylbenzene |
| BRA006 | 180.102 | 9.80 | GNPS2 | ATDWJOOPFDQZNK-UHFFFAOYSA-N | N-[2-(4-hydroxyphenyl)ethyl]acetamide |
| BRA006 | 138.092 | 9.81 | ChemWalker | CDQPLIAKRDYOCB-UHFFFAOYSA-N | 4-(1-aminoethyl)phenol |
| BRA006 | 161.072 | 10.02 | GNPS2 | LSGKMZLPZFPAIN-UHFFFAOYSA-N | 1H-indole-3-carboxamide |
| BRA006 | 116.107 | 11.81 | ChemWalker | ZOFRRNUENOHELM-UHFFFAOYSA-N | 2-amino-4-methylpentanal |
| BRA006 | 185.129 | 11.98 | GNPS2 | JDRIJDPCYNFZIT-ACZMJKKPSA-N | (3S,6S)-3-[(2S)-butan-2-yl]-6-methylpiperazine-2,5-dione |
| BRA006 | 105.070 | 16.60 | ChemWalker | PPBRXRYQALVLMV-UHFFFAOYSA-N | styrene |
| BRA006 | 130.123 | 19.87 | ChemWalker | AEDIXYWIVPYNBI-UHFFFAOYSA-N | heptanamide |
| BRA006 | 164.106 | 19.93 | ChemWalker | LMDWIEAYQGRUHB-UHFFFAOYSA-N | 2-amino-3-phenylbutanal |
| BRA006 | 162.091 | 19.93 | ChemWalker | GLJWZMYXVRYLPB-UHFFFAOYSA-N | 3-(2-methylphenyl)prop-2-enamide |
| BRA006 | 164.107 | 20.33 | ChemWalker | NVKXBDITZDURNJ-UHFFFAOYSA-N | N-[(4-propan-2-ylphenyl)methylidene]hydroxylamine |
| BRA006 | 177.091 | 22.03 | SIRIUS | FDBYIFZQKNMIMY-UHFFFAOYSA-N | 8-fluoro-8-oxooctanoic acid |
| BRA006 | 595.381 | 30.83 | ChemWalker | IZZIZHGPMWXMBD-UHFFFAOYSA-N | 2-[[9,14-dihydroxy-14-(methoxymethyl)-3,10-dimethyl-6-propan-2-yl-8-tricyclo[9.3.0.03,7]tetradeca-1,6-dienyl]oxy]-6-(methoxymethyl)-5-(3-methylbut-1-enoxy)oxane-3,4-diol |
| BRA006 | 411.309 | 32.20 | ChemWalker | OELDKSOVLPNCTH-UHFFFAOYSA-N | 3-henicosa-12,16-dienoyloxy-2-hydroxypropanoic acid |
| BRA010 | 110.060 | 0.65 | ChemWalker | SOHMZGMHXUQHGE-UHFFFAOYSA-N | 5-methyl-1H-pyridin-2-one |
| BRA010 | 108.044 | 0.79 | ChemWalker | CSDSSGBPEUDDEE-UHFFFAOYSA-N | pyridine-2-carbaldehyde |
| BRA010 | 140.082 | 0.80 | ChemWalker | VYOIELONWKIZJS-UHFFFAOYSA-N | 2-amino-3-(1H-imidazol-5-yl)propanal |
| BRA010 | 199.064 | 0.82 | SIRIUS | ZYCRVTMORAHQAH-RITPCOANSA-N | [(2S,3R)-3-chloro-2-(hydroxymethyl)pyrrolidin-1-yl]-methylborinic acid |
| BRA010 | 126.055 | 0,826 | ChemWalker | VJBRLHBYLMMWER-UHFFFAOYSA-N | N-[(5-methylfuran-2-yl)methylidene]hydroxylamine |
| BRA010 | 167.093 | 0.83 | ChemWalker | PKHMCXYXGDYUJF-UHFFFAOYSA-N | 4,5,6,7-tetrahydro-3H-imidazo[4,5-c]pyridine-6-carboxamide |
| BRA010 | 185.042 | 0.89 | SIRIUS | OHCFEDLDHLJTKN-UHFFFAOYSA-N | (4-sulfinamoylphenyl)boronic acid |
| BRA010 | 113.035 | 1.10 | SIRIUS | ISAKRJDGNUQOIC-UHFFFAOYSA-N | 1H-pyrimidine-2,4-dione |
| BRA010 | 140.071 | 2.23 | SIRIUS | UWTVKNXZTGSDDT-UHFFFAOYSA-N | 4,6-dioxoheptanenitrile |
| BRA010 | 155.081 | 2.26 | SIRIUS | JZCWIPSHOQIMCT-UHFFFAOYSA-N | 1-(1-hydroxypropan-2-yl)imidazole-4-carbaldehyde |
| BRA010 | 146.044 | 2.73 | SIRIUS | UNEAFRQQLNNTEP-UHFFFAOYSA-N | O-(2,2,2-trifluoroethoxymethyl)hydroxylamine |
| BRA010 | 111.044 | 2.98 | SIRIUS | QIGBRXMKCJKVMJ-UHFFFAOYSA-N | benzene-1,4-diol |
| BRA010 | 199.108 | 3.03 | ChemWalker | UBLWFFBGMBRBMC-UHFFFAOYSA-N | 3-(1-hydroxyethyl)-2,3,6,7,8,8a-hexahydropyrrolo[1,2-a]pyrazine-1,4-dione |
| BRA010 | 157.097 | 3.07 | SIRIUS | MPUHTIPZENEYSH-UHFFFAOYSA-N | N-methyl-2-oxo-2-pyrrolidin-1-ylacetamide |
| BRA010 | 169.097 | 3.78 | GNPS2 | LEHOTFFKMJEONL-UHFFFAOYSA-N | 7,9-dihydro-3H-purine-2,6,8-trione |
| BRA010 | 130.032 | 4.83 | SIRIUS | ITFMBQKRDMBOON-ORCRQEGFSA-N | |
| BRA010 | 112.022 | 4.84 | SIRIUS | SCFQKZUPNGMPBM-UHFFFAOYSA-N | N-(difluoromethoxy)formamide |
| BRA010 | 196.097 | 6.78 | ChemWalker | OFSAJYZMIPNPHE-UHFFFAOYSA-N | N-[2-(3,4-dihydroxyphenyl)ethyl]acetamide |
| BRA010 | 137.060 | 6.83 | ChemWalker | ZWLPBLYKEWSWPD-UHFFFAOYSA-N | 2-methylbenzoic acid |
| BRA010 | 195.113 | 6.92 | ChemWalker | ASTXJGGHDAJLLS-UHFFFAOYSA-N | 4-methyl-1-(4-methyl-5-oxopyrrolidin-2-yl)-2H-pyrrol-5-one |
| BRA010 | 171.113 | 7.23 | GNPS2 | GINVVVBYRCSQIJ-UHFFFAOYSA-N | 2-(2-oxoazepan-1-yl)acetamide |
| BRA010 | 121.065 | 7.55 | SIRIUS | KWOLFJPFCHCOCG-UHFFFAOYSA-N | 1-phenylethanone |
| BRA010 | 95.086 | 8.36 | SIRIUS | HEKRPWJODWSOQV-UHFFFAOYSA-N | 3-ethenylpenta-1,4-diene |
| BRA010 | 41.091 | 8.37 | SIRIUS | PNTAWGJKWLLAAW-PHDIDXHHSA-N | (1R,6R)-bicyclo[4.1.0]heptane-7-carboxylic acid |
| BRA010 | 138.091 | 9.87 | GNPS2 | DZGWFCGJZKJUFP-UHFFFAOYSA-N | 4-(2-aminoethyl)phenol |
| BRA010 | 103.054 | 9.88 | ChemWalker | UEXCJVNBTNXOEH-UHFFFAOYSA-N | ethynylbenzene |
| BRA010 | 121.065 | 10.17 | ChemWalker | BTFQKIATRPGRBS-UHFFFAOYSA-N | 2-methylbenzaldehyde |
| BRA010 | 197.128 | 10.36 | GNPS2 | LGBLJRRKJGMNEN-UHFFFAOYSA-N | 1-propan-2-yl-1,2,6,7,8,8a-hexahydroimidazo[1,5-a]pyridine-3,5-dione |
| BRA010 | 178.086 | 10.54 | ChemWalker | OXFGRWIKQDSSLY-UHFFFAOYSA-N | 1,2,3,4-tetrahydroisoquinoline-1-carboxylic acid |
| BRA010 | 147.092 | 10.87 | ChemWalker | ZXIXBPGNGUZOBB-UHFFFAOYSA-N | 2-amino-2-phenylpropanenitrile |
| BRA010 | 185.128 | 12.57 | ChemWalker | COAIJFYBVHDGBZ-UHFFFAOYSA-N | 6-pentyl-1,3-diazinane-2,4-dione |
| BRA010 | 185.128 | 12.93 | GNPS2 | DBJPZCJQDRPOME-BQBZGAKWSA-N | (3S,6S)-3-methyl-6-(2-methylpropyl)piperazine-2,5-dione |
| BRA010 | 164.071 | 13.27 | ChemWalker | OWMJAQBUFVTERI-UHFFFAOYSA-N | N-(2-formylphenyl)acetamide |
| BRA010 | 164.071 | 13.83 | ChemWalker | SMIJNYOAPGHFBC-UHFFFAOYSA-N | 3-(hydroxyamino)-2-phenylprop-2-enal |
| BRA010 | 194.118 | 13.92 | ChemWalker | VIFWHZYHQSVJGD-UHFFFAOYSA-N | N-[2-(4-methoxyphenyl)ethyl]acetamide |
| BRA010 | 176.071 | 14.93 | GNPS2 | ZOAMBXDOGPRZLP-UHFFFAOYSA-N | 2-(1H-indol-3-yl)acetamide |
| BRA010 | 105.070 | 16.67 | ChemWalker | PPBRXRYQALVLMV-UHFFFAOYSA-N | styrene |
| BRA010 | 122.097 | 18.76 | SIRIUS | TUZXLRNKEQITEA-UHFFFAOYSA-N | N-but-3-ynylbut-2-yn-1-amine |
| BRA010 | 168.066 | 20.63 | ChemWalker | DQLYTFPAEVJTFM-UHFFFAOYSA-N | 2-amino-2-(3-hydroxyphenyl)acetic acid |
| BRA010 | 197.118 | 24.84 | ChemWalker | PWZHEFFAUWAEMU-UHFFFAOYSA-N | 3-but-2-enyl-4-(2-hydroxypropyl)-2H-furan-5-one |
| BRA010 | 109.065 | 26.84 | SIRIUS | IBZHQYDVHLIBLL-UHFFFAOYSA-N | 2-fluorobutane-1,4-diol |
| BRA010 | 431.277 | 29.12 | ChemWalker | NEZOHSNNESSNOS-UHFFFAOYSA-N | 5-(4-butan-2-yl-1,5-dihydroxy-5,6,9-trimethyl-2,4,5a,7a,8,11,11a,11b-octahydro-1H-benzo[i][3]benzoxepin-2-yl)penta-2,4-dienoic acid |
| BRA010 | 595.382 | 30.84 | ChemWalker | IZZIZHGPMWXMBD-UHFFFAOYSA-N | 2-[[9,14-dihydroxy-14-(methoxymethyl)-3,10-dimethyl-6-propan-2-yl-8-tricyclo[9.3.0.03,7]tetradeca-1,6-dienyl]oxy]-6-(methoxymethyl)-5-(3-methylbut-1-enoxy)oxane-3,4-diol |
| BRA010 | 639.408 | 30.87 | ChemWalker | YMIXNQVKVPYZHL-UHFFFAOYSA-N | 3-hexyl-4,6,8,10,12,14,16,27-octahydroxy-17,28-dimethyl-1-oxacyclooctacosa-17,19,21,23,25-pentaen-2-one |
| BRA010 | 439.204 | 31.89 | SIRIUS | ODGGVBKRXWOVOK-UHFFFAOYSA-N | N-[2-[2-[2-(prop-2-ynoylamino)ethoxy]ethoxy]ethyl]-11-sulfanylundecanamide |
| BRA010 | 369.239 | 31.90 | SIRIUS | JFAFCZZLYDTYHA-UHFFFAOYSA-N | [2-[[5-[(2-boronophenyl)methylamino]pentylamino]methyl]phenyl]boronic acid |
| BRA177 | 96.045 | 0.82 | ChemWalker | GCNTZFIIOFTKIY-UHFFFAOYSA-N | 1H-pyridin-4-one |
| BRA177 | 126.055 | 0.82 | ChemWalker | VJBRLHBYLMMWER-UHFFFAOYSA-N | N-[(5-methylfuran-2-yl)methylidene]hydroxylamine |
| BRA177 | 167.093 | 0.83 | SIRIUS | PVWNFAGYFUUDRC-UHFFFAOYSA-N | N-[(4-amino-2-methylpyrimidin-5-yl)methyl]formamide |
| BRA177 | 136.062 | 0.83 | GNPS2 | GFFGJBXGBJISGV-UHFFFAOYSA-N | 7H-purin-6-amine |
| BRA177 | 140.082 | 0.84 | ChemWalker | VYOIELONWKIZJS-UHFFFAOYSA-N | 2-amino-3-(1H-imidazol-5-yl)propanal |
| BRA177 | 108.045 | 0.84 | ChemWalker | CSDSSGBPEUDDEE-UHFFFAOYSA-N | pyridine-2-carbaldehyde |
| BRA177 | 110.060 | 0.87 | ChemWalker | OUKQTRFCDKSEPL-UHFFFAOYSA-N | 1-methylpyrrole-2-carbaldehyde |
| BRA177 | 129.066 | 1.05 | ChemWalker | LPQUIFIUJKZJRT-UHFFFAOYSA-N | 1-methyl-1,3-diazinane-2,4-dione |
| BRA177 | 113.035 | 1.07 | ChemWalker | JBTGHKUTYAMZEZ-UHFFFAOYSA-N | but-2-ynediamide |
| BRA177 | 123.055 | 1.15 | GNPS2 | DFPAKSUCGFBDDF-UHFFFAOYSA-N | pyridine-3-carboxamide |
| BRA177 | 137.046 | 1.29 | GNPS2 | FDGQSTZJBFJUBT-UHFFFAOYSA-N | 3,7-dihydropurin-6-one |
| BRA177 | 153.041 | 1.48 | GNPS2 | LRFVTYWOQMYALW-UHFFFAOYSA-N | 3,7-dihydropurine-2,6-dione |
| BRA177 | 113.035 | 1.84 | SIRIUS | ISAKRJDGNUQOIC-UHFFFAOYSA-N | 1H-pyrimidine-2,4-dione |
| BRA177 | 127.050 | 1.93 | GNPS2 | RWQNBRDOKXIBIV-UHFFFAOYSA-N | 5-methyl-1H-pyrimidine-2,4-dione |
| BRA177 | 136.062 | 1.94 | ChemWalker | GFFGJBXGBJISGV-UHFFFAOYSA-N | 7H-purin-6-amine |
| BRA177 | 174.055 | 2.04 | ChemWalker | PCPFJNLCUUEVDQ-UHFFFAOYSA-N | 4-oxo-1H-quinoline-2-carbaldehyde |
| BRA177 | 157.097 | 2.22 | GNPS2 | GFFGJBXGBJISGV-UHFFFAOYSA-N | 7H-purin-6-amine |
| BRA177 | 155.081 | 2.23 | ChemWalker | GRDXZRWCQWDLPG-UHFFFAOYSA-N | 1,3,6-trimethylpyrimidine-2,4-dione |
| BRA177 | 111.044 | 2.41 | ChemWalker | UBTPQJCPHOHKMF-UHFFFAOYSA-N | 5-ethylidenefuran-2-one |
| BRA177 | 164.093 | 2.54 | ChemWalker | BVIAOQMSVZHOJM-UHFFFAOYSA-N | N,N-dimethyl-7H-purin-6-amine |
| BRA177 | 160.076 | 2.60 | SIRIUS | WHOOUMGHGSPMGR-UHFFFAOYSA-N | 2-(1H-indol-3-yl)acetaldehyde |
| BRA177 | 199.108 | 2.92 | GNPS2 | UBLWFFBGMBRBMC-UHFFFAOYSA-N | 3-(1-hydroxyethyl)-2,3,6,7,8,8a-hexahydropyrrolo[1,2-a]pyrazine-1,4-dione |
| BRA177 | 157.097 | 3.02 | ChemWalker | QOSQDHSPKMBGMH-UHFFFAOYSA-N | 2-(2-oxopiperidin-1-yl)acetamide |
| BRA177 | 169.097 | 3.73 | GNPS2 | LEHOTFFKMJEONL-UHFFFAOYSA-N | 7,9-dihydro-3H-purine-2,6,8-trione |
| BRA177 | 127.050 | 3.92 | GNPS2 | HDFGOPSGAURCEO-UHFFFAOYSA-N | 1-ethylpyrrole-2,5-dione |
| BRA177 | 125.071 | 3.95 | ChemWalker | GCQHUBANENYTLB-UHFFFAOYSA-N | 2-(1-methylimidazol-4-yl)acetaldehyde |
| BRA177 | 130.032 | 4.77 | ChemWalker | MWSQHVUUIHWHBM-UHFFFAOYSA-N | 2-(1,3-thiazol-5-yl)ethanol |
| BRA177 | 112.040 | 4.84 | ChemWalker | ZLPORNPZJNRGCO-UHFFFAOYSA-N | 3-methylpyrrole-2,5-dione |
| BRA177 | 195.113 | 6.82 | ChemWalker | KIPFMNAHRGYDMB-UHFFFAOYSA-N | 2-(aminomethyl)-5-methoxy-N-methylbenzamide |
| BRA177 | 171.113 | 7.18 | ChemWalker | GINVVVBYRCSQIJ-UHFFFAOYSA-N | 2-(2-oxoazepan-1-yl)acetamide |
| BRA177 | 146.060 | 7.47 | ChemWalker | OLNJUISKUQQNIM-UHFFFAOYSA-N | 1H-indole-3-carbaldehyde |
| BRA177 | 171.112 | 7.55 | SIRIUS | HPHUVLMMVZITSG-UHFFFAOYSA-N | 2-(2-oxopyrrolidin-1-yl)butanamide |
| BRA177 | 103.054 | 9.73 | GNPS2 | RGHHSNMVTDWUBI-UHFFFAOYSA-N | 4-hydroxybenzaldehyde |
| BRA177 | 121.065 | 9.75 | ChemWalker | FUGYGGDSWSUORM-UHFFFAOYSA-N | 4-ethenylphenol |
| BRA177 | 138.091 | 9.75 | GNPS2 | DZGWFCGJZKJUFP-UHFFFAOYSA-N | 4-(2-aminoethyl)phenol |
| BRA177 | 197.128 | 10.25 | GNPS2 | LGBLJRRKJGMNEN-UHFFFAOYSA-N | 1-propan-2-yl-1,2,6,7,8,8a-hexahydroimidazo[1,5-a]pyridine-3,5-dione |
| BRA177 | 169.076 | 11.96 | ChemWalker | JIFVPBALUHDGEP-UHFFFAOYSA-N | 2-[(4-methylphenyl)methylidene]propanedinitrile |
| BRA177 | 185.128 | 11.98 | GNPS2 | COAIJFYBVHDGBZ-UHFFFAOYSA-N | 6-pentyl-1,3-diazinane-2,4-dione |
| BRA177 | 185.128 | 12.78 | GNPS2 | DBJPZCJQDRPOME-BQBZGAKWSA-N | (3S,6S)-3-methyl-6-(2-methylpropyl)piperazine-2,5-dione |
| BRA177 | 164.071 | 13.66 | ChemWalker | SMIJNYOAPGHFBC-UHFFFAOYSA-N | 3-(hydroxyamino)-2-phenylprop-2-enal |
| BRA177 | 176.071 | 14.91 | GNPS2 | ZOAMBXDOGPRZLP-UHFFFAOYSA-N | 2-(1H-indol-3-yl)acetamide |
| BRA177 | 91.054 | 17.04 | ChemWalker | AMSMVCOBCOZLEE-UHFFFAOYSA-N | bicyclo[4.1.0]hepta-1,3,5-triene |
| BRA177 | 137.060 | 17.11 | GNPS2 | RWZYAGGXGHYGMB-UHFFFAOYSA-N | 2-aminobenzoic acid |
| BRA177 | 122.097 | 18.47 | GNPS2 | WPYMKLBDIGXBTP-UHFFFAOYSA-N | benzoic acid |
| BRA177 | 105.070 | 18.71 | ChemWalker | PPBRXRYQALVLMV-UHFFFAOYSA-N | styrene |
| BRA177 | 164.107 | 18.73 | GNPS2 | MODKMHXGCGKTLE-UHFFFAOYSA-N | N-(2-phenylethyl)acetamide |
| BRA177 | 187.089 | 21.09 | ChemWalker | CXBYCIKWTYCLAR-UHFFFAOYSA-N | 1-methyl-5-phenylpyrimidin-2-one |

Supplementary Table 2. Fragmented ions belonging exclusively to the list (SPL) that presented the annotation and their respective metabolic pathways.

| Sample | Compound (IUPAC name) | Metabolic pathway (KEGG) | KEGG compound ID | KEGG pathway ID |
| --- | --- | --- | --- | --- |
| BRA006 | 2-methylbenzaldehyde | Xylene degradation | cpd:C07214 | map00622 |
| BRA006 | benzamide | Aminobenzoate degradation | cpd:C09815 | map00627 |
| BRA006 | 3,7-dihydropurine-2,6-dione | Purine metabolism | cpd:C00385 | map00230 |
| BRA006 | 3-(1H-imidazol-5-yl)prop-2-enoic acid | Histidine metabolism | cpd:C00785 | map00340 |
| BRA006 | 2-(2-hydroxyphenyl)acetic acid | Phenylalanine metabolism | cpd:C05852 | map00360 |
| BRA006 | styrene | Ethylbenzene degradation | cpd:C07083 | map00642 |
| BRA006 | 4-hydroxy-3-methoxybenzoic acid | Aminobenzoate degradation | cpd:C06672 | map00627 |
| BRA006 | 2-(1H-indol-3-yl)acetaldehyde | Tryptophan metabolism | cpd:C00637 | map00380 |
| BRA006 | N-[(4-amino-2-methylpyrimidin-5-yl)methyl]formamide | Thiamine metabolism | cpd:C19872 | map00730 |
| BRA010 | 1H-pyrimidine-2,4-dione | Pyrimidine metabolism | cpd:C00106 | map00240 |
| BRA010 | (i) 4-(2-aminoethyl)phenol ; (ii) benzene-1,4-diol | Tyrosine metabolism | (i) cpd:C00483; (ii) cpd:C00530 | map00350 |
| BRA010 | styrene | Styrene degradation | cpd:C07083 | map00643 |
| BRA010 | 2-(1H-indol-3-yl)acetamide | Tryptophan metabolism | cpd:C02693 | map00380 |
| BRA010 | (i) 2-methylbenzoic acid; (ii) 2-methylbenzaldehyde | Xylene degradation | (i) cpd:C07215; (ii) cpd:C07214 | map00622 |
| BRA010 | 2-amino-3-(1H-imidazol-5-yl)propanal | Histidine metabolism | cpd:C01929 | map00340 |
| BRA010 | 1-phenylethanone | Ethylbenzene degradation | cpd:C07113 | map00642 |
| BRA010 | 7,9-dihydro-3H-purine-2,6,8-trione | Purine metabolism | cpd:C00366 | map00230 |
| BRA177 | (i) 7H-purin-6-amine; (ii) 3,7-dihydropurin-6-one; (iii) 3,7-dihydropurine-2,6-dione; (iv) 7,9-dihydro-3H-purine-2,6,8-trione | Purine metabolism | (i) cpd:C00147; (ii): cpd:C00262; (iii) cpd:C00385; (iv) cpd:C00366 | map00230 |
| BRA177 | 4-ethenylphenol | Phenylpropanoid biosynthesis | cpd:C05627 | map00940 |
| BRA177 | 4-(2-aminoethyl)phenol | Tyrosine metabolism | cpd:C00483 | map00350 |
| BRA177 | styrene | Styrene degradation | cpd:C07083 | map00643 |
| BRA177 | (i) 5-methyl-1H-pyrimidine-2,4-dione; (ii) 1H-pyrimidine-2,4-dione | Pyrimidine metabolism | (i) cpd:C00178; (ii) cpd:C00106 | map00240 |
| BRA177 | N-[(4-amino-2-methylpyrimidin-5-yl)methyl]formamide | Thiamine metabolism | cpd:C19872 | map00730 |
| BRA177 | (i) 2-(1H-indol-3-yl)acetaldehyde; (ii) 2-aminobenzoic acid; (iii) 2-(1H-indol-3-yl)acetamide | Tryptophan metabolism | (i) cpd:C00637; (ii) cpd:C00108; (iii) cpd:C02693 | map00380 |
| BRA177 | (i) 2-(1-methylimidazol-4-yl)acetaldehyde; (ii) 2-amino-3-(1H-imidazol-5-yl)propanal | Histidine metabolism | (i) cpd:C05827; (ii) cpd:C01929 | map00340 |
| BRA177 | 4-hydroxybenzaldehyde | Aminobenzoate degradation | cpd:C00633 | map00627 |
| BRA177 | pyridine-3-carboxamide | Nicotinate and nicotinamide metabolism | cpd:C00153 | map00760 |
| BRA177 | benzoic acid | Benzoate degradation | cpd:C00180 | map00362 |
